# Literary Notes

**Published:** 1901-03

**Authors:** 


### LITERARY NOTES.

THE Texas *Medical Gazette* made its appearance in January of the present year. It is printed at Fort Worth, and edited by Drs. F. D. Thompson, Bacon Saunders, Frank Gray and W. R. Thompson. The first number contains considerable interesting original material, besides a number of editorials on current topics.

---

*The Stylus* has been consolidated with the *Interstate Medical Journal* and the two publications continued under the latter name. Dr. William Porter, formerly editor of *The Stylus*, will be associated with Drs. W. B. Outten, R. B. H. Gradwohl and O. F. Ball, in the editorial management of the *Interstate Medical Journal*, St. Louis, Mo.

---

*American Medicine*, the new weekly projected and promoted by Dr. George M. Gould, who was so forcibly and suddenly ejected from the Philadelphia *Medical Journal* a few weeks ago, will make its appearance either late in March or early in April at farthest. It has already taken solid financial footing, and it goes without saying that its editorial side with Dr. Gould at the head will be of the best. We have no doubt this new journalistic enterprise will prove an ornament to American periodical literature, as well as a profitable enterprise for its owners.

---

PRIZE ESSAY ON THE DANGERS FROM QUACKERY.—The Colorado State Medical Society offers a prize of $25.00 for the best essay, if deemed worthy of the prize, pointing out the dangers to public health and morals, especially to young persons, from quackery as promulgated by public advertisements.

The competition is open to all. Essays must be typewritten in the English language, and submitted before May 15, 1901. Each essay must be designated by a motto and accompanied by a sealed envelope, bearing the same motto, and enclosing the name and address of the author. The essay receiving the prize will become the property of the society for publication. Others will be returned on application. Essays should be sent to the Literature Committee, Room 315 McPhee Building, Denver, Colorado.

---

THE Pan-American Exposition Company, through its Bureau of Publicity, has just issued another beautiful booklet, illustrating some of the many interesting features that are planning for the instruction and amusement of the visitors. It consists of 16 pages covered in light green with a unique miniature reproduction of the famous poster, "The Spirit of Niagara." It is a beautiful picture-book containing fine views of all the principal buildings and giving many scenes that will take place during the exhibition.

Among other things the center of the booklet shows a birdseye view of the exposition, and gives one some idea of the great extent of the enterprise upon which about $10,000,000 is being expended. The grounds contain 350 acres, being half a mile wide, and a mile and a quarter long. Other pages show horticulture, graphic arts and mines, manufactures and liberal arts, the music temple, the plaza and its beautiful surroundings, the stadium or athletic field, the agricultural, live stock and ethnology features, and a few of the thirty or forty ingenious and novel exhibits, which promise to make the midway the most wonderful that has ever been prepared for exposition visitors. The last page shows a ground plan of the exposition, whereon the location of different buildings is indicated. The railroads will make low rates from all parts of the country during the exposition, which opens May 1, 1901, and continues six months. As the date approaches the interest in the fair increases, and it has already grown in scope and size beyond the original plans of its projectors. The people of Buffalo are preparing to entertain comfortably the millions who will attend. Anyone desiring a copy of this booklet may have it free by addressing the Pan-American Bureau of Publicity.

